# Comparative CKD risk prediction using homocitrulline and carbamylated albumin: two circulating markers of protein carbamylation

**DOI:** 10.1186/s12882-024-03619-6

**Published:** 2024-05-30

**Authors:** Aya Awwad, Eugene P. Rhee, Morgan Grams, Hernan Rincon Choles, James Sondheimer, Jiang He, Jing Chen, Chi-yuan Hsu, Ramachandran S Vasan, Paul L. Kimmel, Kendra Wulczyn, Anders Berg, Jim Lash, Mengyao Tang, Sahir Kalim, the CRIC Study Investigators, Amanda H Anderson, Lawrence J. Appel, Debbie L Cohen, Laura M Dember, Alan S. Go, Robert G. Nelson, Mahboob Rahman, Panduranga S. Rao, Vallabh O Shah, Mark L. Unruh

**Affiliations:** 1 Department of Medicine, Division of Nephrology, Massachusetts General Hospital, Harvard Medical School, Boston, MA USA; 2 Department of Medicine, New York University, New York, NY USA; 3 Department of Nephrology, Glickman Urological and Kidney Institute, Cleveland Clinic, Cleveland, OH USA; 4 Department of Medicine, Wayne State University, Detroit, MI USA; 5 Department of Epidemiology, Tulane University School of Public Health and Tropical Medicine, New Orleans, LA USA; 6 Department of Medicine, Tulane University School of Medicine, New Orleans, LA USA; 7 Division of Nephrology, University of California San Francisco School of Medicine, San Francisco, CA USA; 8 Division of Research, Kaiser Permanente Northern California, Oakland, CA USA; 9 Department of Epidemiology, Boston University School of Public Health, Boston, MA USA; 10 Department of Medicine, Sections of Preventive Medicine and Epidemiology and Cardiology, Boston University School of Medicine, Boston, MA USA; 11 Division of Kidney, Urologic, and Hematologic Diseases, National Institute of Diabetes and Digestive and Kidney Diseases (NIDDK), Bethesda, MD USA; 12 Department of Pathology and Laboratory Medicine, Cedars-Sinai Medical Center, Los Angeles, CA USA; 13 Department of Medicine, University of Illinois at Chicago, Chicago, IL USA

**Keywords:** Biomarker, Carbamylation, Carbamylated albumin, Chronic kidney disease, Homocitrulline

## Abstract

**Background:**

Protein carbamylation, a post-translational protein modification primarily driven by urea, independently associates with adverse clinical outcomes in patients with CKD. Biomarkers used to quantify carbamylation burden have mainly included carbamylated albumin (C-Alb) and homocitrulline (HCit, carbamylated lysine). In this study, we aimed to compare the prognostic utility of these two markers in order to facilitate comparisons of existing studies employing either marker alone, and to inform future carbamylation studies.

**Methods:**

Both serum C-Alb and free HCit levels were assayed from the same timepoint in 1632 individuals with CKD stages 2–4 enrolled in the prospective Chronic Renal Insufficiency Cohort (CRIC) study. Adjusted Cox proportional hazard models were used to assess risks for the outcomes of death (primary) and end stage kidney disease (ESKD) using each marker. C-statistics, net reclassification improvement, and integrated discrimination improvement were used to compare the prognostic value of each marker.

**Results:**

Participant demographics included mean (SD) age 59 (11) years; 702 (43%) females; 700 (43%) white. C-Alb and HCit levels were positively correlated with one another (Pearson correlation coefficient 0.64). Higher C-Alb and HCit levels showed similar increased risk of death (e.g., the adjusted hazard ratio [HR] for death in the 4th carbamylation quartile compared to the 1st was 1.90 (95% confidence interval [CI] 1.35–2.66) for C-Alb, and 1.89 [1.27–2.81] for HCit; and on a continuous scale, the adjusted HR for death using C-Alb was 1.24 [1.11 to 1.39] per standard deviation increase, and 1.27 [1.10–1.46] using HCit). Both biomarkers also had similar HRs for ESKD. The C-statistics were similar when adding each carbamylation biomarker to base models (e.g., for mortality models, the C-statistic was 0.725 [0.707–0.743] with C-Alb and 0.725 [0.707–0.743] with HCit, both compared to a base model 0.723). Similarities were also observed for the net reclassification improvement and integrated discrimination improvement metrics.

**Conclusions:**

C-Alb and HCit had similar performance across multiple prognostic assessments. The markers appear readily comparable in CKD epidemiological studies.

**Supplementary Information:**

The online version contains supplementary material available at 10.1186/s12882-024-03619-6.

## Background

**Fig. 1:**
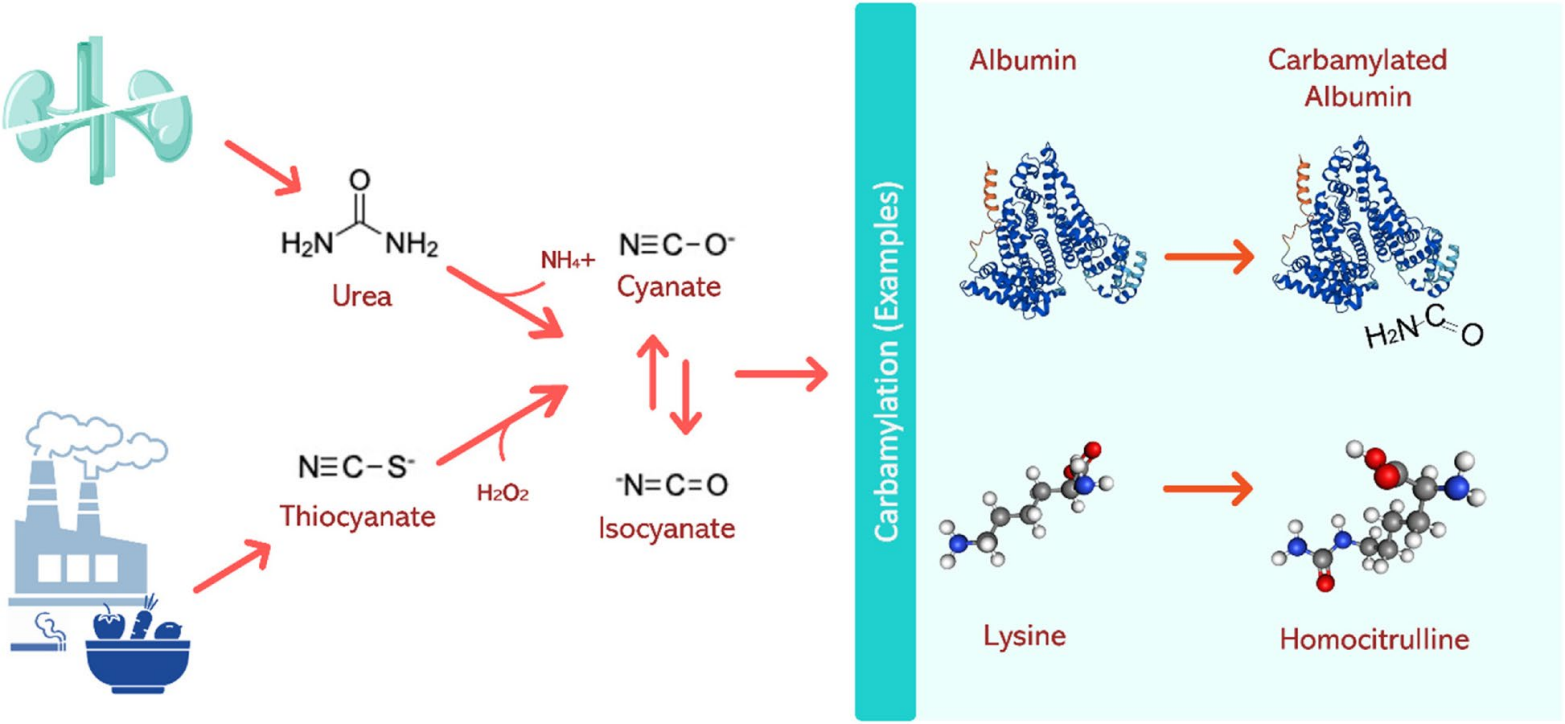
Illustration of the carbamylation process, a systemic post-translational protein modification

Protein carbamylation is a non-enzymatic, post translational protein modification that occurs when cyanate, the dissociation product of urea, spontaneously reacts to bind primary amino groups on proteins (Fig. 1), resulting in protein charge and conformational changes that can impact numerous molecular and cellular functions [1–3]. While carbamylated proteins are found in relatively low concentrations in healthy humans, impaired kidney function leads to elevated blood urea levels and, subsequently, higher levels of carbamylation [1]. Carbamylation in patients with chronic kidney disease (CKD) has been linked to several distinct pathological pathways. For example, carbamylation of select proteins can accelerate the biochemical events of atherosclerosis, trigger vascular calcification, and initiate inflammatory and profibrogenic signaling cascades [4–12].

Epidemiological studies have shown that carbamylation is an independent risk factor for mortality and CKD progression [13–21]. Despite growing attention to carbamylation as a marker of and potential therapeutic target for adverse outcomes in CKD, the optimal assay to measure carbamylation burden remains to be determined. Different assays that have previously been employed include carbamylated protein measures (e.g., carbamylated albumin, C-Alb) as well as direct measures of carbamylated lysine (i.e., homocitrulline, HCit) [1]. HCit itself can be assayed in different forms, namely free and protein bound. Free lysine can become carbamylated to form free HCit, and protein-bound lysine can become carbamylated on its side chain to become protein bound HCit. Notably, the protein bound form is released during in vivo protein degradation generating additional free HCit, thus free HCit levels may reflect the amount of total system carbamylation that is occurring (as well as clearance of this non-proteinogenic amino acid). Free HCit is a commonly measured analyte on widely used metabolomic platforms and is what “HCit” is referring to herein [21].

Non-uniformity in carbamylation assay selection across studies raises challenges for meaningful comparisons across distinct clinical research cohorts. A recent small scale brief report suggested that measures of HCit and C-Alb were positively correlated as expected but clinical outcomes were not assessed [22]. Establishing the comparability of HCit and C-Alb in relation to clinical outcomes stands to rapidly advance carbamylation research by facilitating use of numerous existing databases with HCit data and allowing comparisons of existing studies. Such information could meaningfully advance investigations of the causes, consequences, and potential treatments of carbamylation.

Two different large scale studies recently measured C-Alb and HCit among participants of the Chronic Renal Insufficiency Cohort (CRIC) study, providing a unique opportunity to now compare head-to-head the operating characteristics and prognostic utility of two of the most widely used markers of carbamylation in clinical investigation [20, 21]. In the present study, we aimed to test the hypothesis that baseline levels of C-Alb and HCit demonstrate similar operating characteristics: Specifically, we postulated that the adjusted hazard ratios, when linked to the clinical endpoints of mortality and progression to end stage kidney disease (ESKD), would be similar. Additionally, we hypothesized that these two markers would show similar discrimination metrics when added to models assessing these outcomes.

## Methods

### Study Population

CRIC is a multicenter, prospective observational cohort study [23, 24]. Between June 1, 2003, to September 30, 2008, a total of 3939 individuals, aged 21–74 years old, with estimated glomerular filtration rates (eGFR) of 20–70 mL/min/1.73m^2^, were recruited across 7 US clinical centers in the initial enrollment phase. CRIC study participants completed annual clinic visits and semiannual telephone contact. Individuals with polycystic kidney disease, prior treatment with dialysis, organ transplant, or on active immunosuppressive agents for glomerulonephritis were excluded. All participants in the study provided written consent during enrollment and the study protocol was approved by the institutional review boards at each clinical center.

### Exposures and covariates

The primary exposures of interest were C-Alb and HCit. Both markers were assayed using CRIC serum or plasma samples from the year 1 CRIC study visit (one year after date of enrollment) which were selected due to sample availability [20, 21]. In our study, the year 1 visit is considered “baseline”. C-Alb was measured in 3,111 randomly selected individuals with sample availability as previously described [20]. The measure of C-Alb using mass spectrometry and its analytical validation have been reported before [17]. C-Alb was measured as the ratio of millimoles of carbamylated albumin per mole of total albumin (analogous to the measurement of percentage glycated hemoglobin) [25].

HCit was measured as part of a larger metabolomic study [21]. 1,800 individuals with available samples were randomly selected and underwent blood metabolomic profiling using the Broad Institute Metabolomics Platform. Detailed methods (including characterization of technical and intrapersonal analyte variation among individuals with CKD) from the Broad institute have been previously described [26]. In brief, 10 µL of plasma was extracted with 90 µL of 74.9:24.9:0.2 v/v/v acetonitrile/methanol/formic acid, and the supernatant following centrifugation was separated on a 150 × 2 mm Atlantis HILIC column (Waters). Mass spectrometry analyses were carried out using electrospray ionization in the positive ion mode on a Q Exactive Plus orbitrab mass spectrometer (Thermo Fisher Scientific). In the present study, HCit is a measure of the free form of homocitrulline (i.e., not carbamylated lysine residues within a protein’s amino acid sequence). 1632 individuals had both C-Alb and HCit data available from the same “baseline” timepoint. A more detailed flowchart illustrating the participant selection process is provided in Supplementary Fig. 1.

Sociodemographic characteristics, medical history, lifestyle behaviors, and other clinical data were obtained at the baseline visit for all included participants. Routine laboratory measurements using standard assays were also previously performed on samples from the baseline visit. The eGFR was calculated using the CKD-EPI Creatinine Equation. While the optimal measure to estimate GFR has been debated, we have shown carbamylation associations in CRIC using the equation employed in this study to be robust across numerous different estimations (incorporating both serum creatinine and cystatin C, and excluding race as a variable) [20].

### Definition of outcomes

Our primary outcome was all cause mortality. Our secondary outcome was ESKD, which was defined as receiving long-term dialysis or a kidney transplant. For analysis of the primary outcome, follow up was censored if the participants withdrew, were lost to follow-up, or reached the end of the follow-up period (mid-2017); for the secondary outcome, patients were also censored if they died. Deaths were ascertained from next of kin, retrieval of death certificates or obituaries, review of hospital records, and linkage with the social security death master file. Ascertainment of ESKD was done through semiannual surveillance by the CRIC Study personnel supplemented by cross-linkage with the US Renal Data System (USRDS).

### Statistical analysis

Baseline characteristics were summarized using mean (standard deviations) or median (interquartile ranges) for continuous variables and counts (proportions) for categorical variables, comparing them using parametric or nonparametric tests as appropriate. Due to the right skewed distribution of HCit and C-Alb, the markers were log transformed. Further, to address the difference in units between C-Alb and HCit, we applied a z-score transformation (centering each observation to the mean and scaling by the standard deviation). This normalization process ensures that both biomarkers are on a common scale, allowing for direct comparison. To establish robustness and aid in the interpretability of our findings, we also conducted analyses after categorizing individuals into quartiles according to the baseline levels of each biomarker. Box plots were constructed to visually represent the distribution of each biomarker’s levels within these quartiles.

The Pearson correlation coefficient between the two biomarkers and between each biomarker and blood urea nitrogen (BUN) levels were evaluated. Locally weighted scatterplot smoothing lines were also used to further assess the relationship between the two biomarkers. We ran analyses in parallel using the two different biomarkers (C-Alb or HCit) using unadjusted and then multivariable adjusted linear regression models to examine the association between the levels of each biomarker and select clinical variables.

We then examined the risk of the primary and secondary outcomes using multivariate adjusted Cox proportional hazards regression models. Because death precludes the occurrence of future ESKD, we used competing risk regression for the ESKD end point as a sensitivity analysis. Covariates for the multivariable adjusted models were selected a priori based on established risks for the outcomes and clinical and biological plausibility of covariates’ potentially confounding the association between carbamylation and the outcomes of interest. The adjusted models were stratified by clinical center and include age, sex, race and ethnicity, systolic blood pressure, body mass index, smoking status, history of diabetes, cardiovascular disease, use of angiotensin-converting enzyme inhibitor or angiotensin II receptor blocker medications, serum total albumin, eGFR, log-transformed 24-hour proteinuria, and reported cause of kidney disease. Given its known association to carbamylation, to assess urea’s impact on our findings, we added blood urea nitrogen (BUN) levels to the models in a sensitivity analysis. Additionally, we reanalyzed the data using the HCit/ total free lysine ratio as a carbamylation biomarker as this ratio has also been employed in prior studies. We finally conducted a stratified analysis based on eGFR levels.

We evaluated the improvement in model performance by the inclusion of each carbamylation marker to a base model adjusted for all pre-specified covariates noted above in the study sample using various metrics [27]: We used Gönen and Heller modified *C*-statistics to compare model discrimination [28], “category-free” net reclassification improvement (NRI) to assess the ability of the model to correctly reclassify risk groups, and integrated discrimination improvement (IDI) [27] to examine the ability of the model to increase average sensitivity without reducing average specificity. Only 0.6% of all data points were missing, where urinary protein had 8% missingness, hemoglobin 1%, and blood urea nitrogen < 0.1%. The rest of the variables were complete. For the primary analysis we conducted a complete case analysis, complimented by multiple imputation using predictive mean matching as a sensitivity analysis. Statistical tests were two sided and *p*-values < 0.05 were considered statistically significant. All analyses were performed in R studio, version 4.2.2.

## Results

### Baseline characteristics

The baseline characteristics of the study population are shown in Table 1, displayed according to the 1st and 4th biomarker quartiles. Supplementary Tables 1 and 2 shows baseline characteristics by all biomarker quartiles. The mean age of the overall study population was 59 (11) years, with 43% females and 43% self-reported white race individuals. At baseline, the overall population mean eGFR was 42 (16) ml/min/1.73 m^2^, BUN level 32 (16) mg/dL, and 24-hour urine protein 0.17 [0.07, 0.92] g/24 h. BUN was consistently higher in the higher carbamylation quartiles (for C-Alb in the 4th quartile, BUN 48 (19) mg/dL, for HCit in the 4th quartile BUN 49 (19) mg/dL. Supplementary Fig. 2 illustrates box plots depicting the distribution of each biomarker across each quartile.

**Table 1:** Baseline characteristics of the study participants according to carbamylated albumin quartile and homocitrulline quartile

		Carbamylated albumin		Homocitrulline	
	Overall	1st quartile	4th quartile	1st quartile	4th quartile
n	1,632	408	408	408	408
Age, years	59 (11.0)	55 (10.7)	60 (11.0)	57 (10.7)	59 (11.3)
**Sex, No (%)**					
Female	702 (43)	183 (45)	171(42)	166 (41)	194 (48)
**Race/Ethnicity, No (%)**					
Non-Hispanic White	700 (43)	170 (42)	164 (40)	215 (53)	119 (29)
Non-Hispanic Black	690 (42)	186 (46)	174 (43)	157 (39)	199 (49)
Hispanic	187 (12)	35 (9)	59 (15)	25 (6)	79 (19)
Other	55 (3)	17 (4)	11 (3)	11 (3)	11 (3)
**Past Medical History, No (%)**					
Hypertension	1466 (90)	334 (82)	385 (95)	324 (80)	391 (96)
Diabetes	816 (50)	180 (44)	236 (58)	120 (29)	273 (67)
CHF	182 (11)	32 (8)	74 (18)	21 (5)	82 (20)
Stroke	182 (11)	32 (8)	52 (13)	29 (7)	52 (13)
PVD	123 (8)	15 (4)	53 (13)	13 (3)	46 (11)
Current Smoking	197 (12)	54 (13)	44 (11)	37 (9)	48 (12)
BMI (mean (SD))	32 (7.7)	34 (7.4)	31 (7.8)	32 (7.2)	33 (8.0)
SBP, mmHg (mean (SD))	130 (20.9)	130 (20.9)	130 (22.7)	120 (18.5)	130 (23.0)
**Medication use, No (%)**					
Aspirin	774(48)	171 (42)	210 (52)	174 (43)	206 (51)
Beta blocker	844 (52)	187 (46)	245 (61)	166 (41)	261 (64)
Statins	970 (60)	214 (53)	271 (67)	192 (47)	275 (68)
ACE or ARB	1139 (70)	247 (61)	304 (75)	239 (59)	299 (74)
**Laboratory data**					
Serum creatinine, mg/dL	2.0 (1.0)	1.5 (0.5)	2.8 (1.3)	1.4 (0.4)	2.9 (1.3)
eGFR ml/min/1.73 m^2^	42 (16.1)	55 (15.4)	30 (11.8)	58 (13.9)	27 (10.9)
Urinary protein, g/ 24 h	0.17 [0.07,0.92]	0.12 [0.06,0.51]	0.33 [0.10,1.16]	0.09 [0.05,0.20]	0.56 [0.15,1.87]
Blood urea nitrogen, mg/dL	32 (16.0)	20 (6.8)	48 (19.2)	20 (6.6)	49 (18.5)
Serum albumin, g/dL	4.0 (0.4)	4.1 (0.4)	4.0 (0.4)	4.2 (0.4)	3.9 (0.5)
Hemoglobin, g/dL	13 (1.8)	14 (1.6)	12 (1.6)	14 (1.6)	12 (1.6)

Values for continuous variables are presented as mean (SD) or median [interquartile range], unless otherwise noted

*Abbreviations*: *No* Number, *CHF* Congestive heart failure, *PVD* Peripheral vascular disease, *BMI* Body mass index (calculated as weight in kilograms divided by height in meters squared), *SBP* Systolic blood pressure, *ACE* Angiotensin-converting enzyme inhibitor, *ARB* Angiotensin II receptor blocker, *eGFR* Estimated glomerular filtration rate

**Fig. 2:**
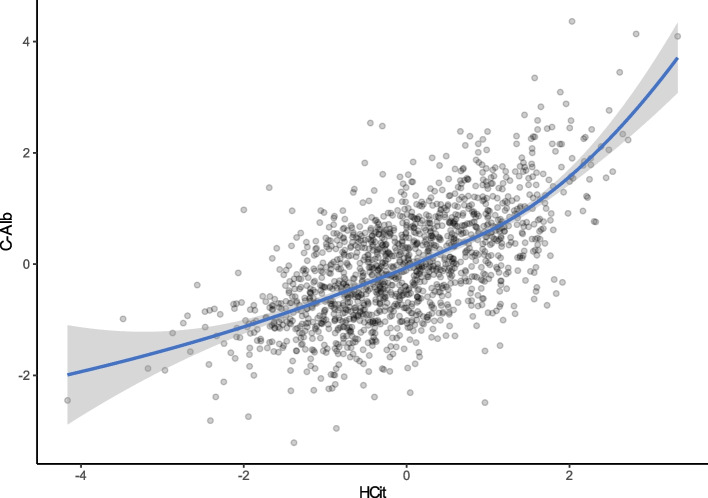
Correlation between carbamylated albumin (C-Alb) and homocitrulline (HCit)

The correlation coefficient between the two log transformed biomarkers was 0.64 (*p* value < 0.001; Fig. 2). Table 2 shows the different factors associated with C-Alb and HCit levels in univariable and multivariable linear regression analysis. Of note, the strongest association observed for either biomarker was BUN, which also showed a correlation coefficient of 0.72 (*p* value < 0.001) for C-Alb, and 0.77 (*p* value < 0.001) for HCit (Supplementary Table 3). Supplementary Fig. 3 shows that the low to high quartiles of each marker were highly concordant to the other marker.

**Table 2:** Factors associated with carbamylated albumin and homocitrulline levels in univariable and multivariable linear regression analysis

	Univariable regression				Multivariable regression			
	Carbamylated albumin		Homocitrulline		Carbamylated albumin		Homocitrulline	
	β Coefficient	*p* value	β Coefficient	*p* value	β Coefficient	*p* value	βCoefficient	*p* value
Age (per year)	0.02	< 0.001	0.01	0.001	0.01	0.004	0.00	0.015
Female (vs. male)	-0.08	0.130	0.06	0.226	-0.11	0.005	0.04	0.252
Black race (vs. white)	0.01	0.803	0.26	< 0.001	-0.10	0.007	0.07	0.036
Hispanic	0.28	0.002	0.70	< 0.001	-0.08	0.174	0.23	< 0.001
Other race	-0.21	0.144	0.10	< 0.001	-0.18	0.051	0.07	0.399
Diabetes	0.20	< 0.001	0.56	< 0.001	-0.24	< 0.001	0.08	0.021
CVD	0.24	< 0.001	0.35	< 0.001	0.01	0.877	0.06	0.070
Systolic blood pressure (per mmHg)	0.00	0.014	0.01	< 0.001	0.00	0.180	0.00	0.311
Body mass index (per 1 unit)	-0.02	< 0.001	0.01	0.063	-0.02	< 0.001	0.00	0.295
Smoking status	-0.09	0.259	0.03	0.698	0.01	0.815	0.02	0.616
eGFR (per mL/min/1.73m2)	-0.04	< 0.001	-0.05	< 0.001	-0.007	< 0.001	-0.02	< 0.001
Urinary protein (per g/ 24 h)^a^	0.07	< 0.001	0.20	< 0.001	-0.07	< 0.001	-0.003	0.796
Serum albumin (per g/dL)	-0.17	0.004	-0.49	< 0.001	0.05	0.250	-0.05	0.261
Hemoglobin (per g/dL)	-0.21	< 0.001	-0.23	< 0.001	-0.12	< 0.001	-0.04	< 0.001
Blood urea nitrogen (per mg/dL)	1.57	< 0.001	1.69	< 0.001	1.39	< 0.001	1.07	< 0.001

Multivariable models included all other variables in the table

*Abbreviations*: *CVD* Cardiovascular disease, *eGFR* Estimated glomerular filtration rate

^a^Urinary protein is natural log scaled

### Outcome analysis

Over a mean follow-up time of 10.6 (3.4) years, there were 494 deaths and 436 ESKD events. Table 3 displays the results of both the unadjusted and the adjusted multivariable Cox proportional hazard model for the two biomarkers, modeled continuously and in quartiles, across both the primary and secondary end points. The adjusted HR for death in quartile 4 compared to 1, was 1.90 (1.35–2.66) for C-Alb, and 1.89 (1.27–2.81) for HCit. On a continuous scale, each SD increase in C-Alb was associated with a 24% higher risk of mortality (HR 1.24; 95% CI 1.11 to 1.39), and each SD increase in HCit was associated with a 27% higher risk of mortality (HR 1.27; 95% CI 1.10 to 1.46). For the secondary endpoint of progression to ESKD, the adjusted HR in quartile 4 compared to 1 was 2.16 (1.47–3.19) for C-Alb, and 2.92 (1.76–4.83) for HCit. On a continuous scale, each SD increase in C-Alb was associated with a 44% higher risk of ESKD (HR 1.44; 95% CI 1.27 to 1.63), and each SD increase in HCit was associated with a 59% higher risk of mortality (HR 1.59; 95% CI 1.36 to 1.86). Supplementary Tables 4 and 5 display additional models with adjustment for BUN. For both markers, the adjustment only modestly attenuated the HR for both endpoints. Supplementary Tables 6,7,8,9 and 10 display the same analysis while modelling HCit as a ratio to total lysine, with largely similar results. Moreover, multiple imputation for missing covariate values was performed in a sensitivity analysis, and no substantial differences were observed. Supplementary Tables 11, 12 display the results of a competing risk analysis and stratified analysis by eGFR, demonstrating stable and comparable performance of the two biomarkers.

**Table 3:** Risk of ESKD and death across the different carbamylation biomarkers

Hazard Ratio (95% confidence interval)				
	Carbamylated albumin		Homocitrulline	
	Unadjusted	Adjusted model^a^	Unadjusted	Adjusted model^a^
**Death**				
Biomarker, per 1-SD increase	1.48 (1.37–1.61)	1.24 (1.11–1.39)	1.62 (1.48–1.76)	1.27 (1.10–1.46)
Quartile 1	*Reference*	*Reference*	*Reference*	*Reference*
Quartile 2	1.35 (1.01–1.81)	1.22 (0.90–1.66)	1.88 (1.38–2.56)	1.19 (0.86–1.65)
Quartile 3	1.99 (1.51–2.62)	1.54 (1.13–2.10)	2.83 (2.10–3.80)	1.65 (1.18–2.32)
Quartile 4	2.99 (2.29–3.90)	1.90 (1.35–2.66)	3.98 (2.99–5.29)	1.89 (1.27–2.81)
**ESKD**				
Biomarker, per 1-SD increase	2.06 (1.87–2.27)	1.44 (1.27–1.63)	2.85 (2.57–3.16)	1.59 (1.36–1.86)
Quartile 1	*Reference*	*Reference*	*Reference*	*Reference*
Quartile 2	1.95 (1.38–2.74)	1.39 (0.96–2.02)	3.49 (2.24–5.44)	1.43 (0.90–2.29)
Quartile 3	3.11 (2.24–4.31)	1.84 (1.27–2.67)	6.46 (4.22–9.89)	1.83 (1.14–2.95)
Quartile 4	5.72 (4.18–7.83)	2.16 (1.47–3.19)	18.23 (12.11–27.44)	2.92 (1.76–4.83)

*Abbreviations*: *ESKD* End stage kidney disease, *SD* Standard deviation

^a^Adjusted model is stratified by center and adjusts for age, sex, race, and ethnicity, systolic blood pressure, body mass index, smoking status, history of diabetes, cardiovascular disease, use of angiotensin-converting enzyme inhibitor or angiotensin II receptor blocker medications, serum total albumin, estimated glomerular filtration rate, natural log-transformed proteinuria, and cause of kidney disease

Finally, we compared the markers using measures of discrimination and reclassification. When adding each carbamylation marker to the adjusted base models for the outcomes of death or ESKD, we found the markers demonstrated similar incremental prognostic value. For example, including C-Alb to the base model predicting mortality yielded a C-statistic of 0.725 (0.707–0.743) whereas including HCit yielded a C-statistic of 0.725 [0.707–0.743], compared to the base model alone C-statistic of 0.723 (Table 4). Addition of carbamylation markers to the base model predicting mortality led to a significant improvement in classification accuracy with a category free NRI of 0.495 (0.112–0.640, *p* value 0.04) for C-Alb vs. 0.519 (0.310–0.726, *p* value 0.03) for HCit, and IDI of 0.010 (-0.004-0.032, *p* value 0.08) for C-Alb vs. 0.010 (0-0.036, *p* value 0.08) for HCit (Table 5). Results were similar for the ESKD outcome (Table 5).

**Table 4:** Comparison of C-statistic values with the addition of each carbamylation biomarker to a base model

C-statistic	Base model^a^	Base model + carbamylated albumin	Base model + homocitrulline
**Death**	0.723 (0.704, 0.741)]	0.725 (0.707, 0.743)	0.725 (0.707, 0.743)
**ESKD**	0.830 (0.818, 0.842)	0.834 (0.822, 0.846)	0.836 (0.825, 0.848)

*Abbreviations*: *ESKD* End stage kidney disease

^a^Base model is stratified by center and adjusts for age, sex, race, and ethnicity, systolic blood pressure, body mass index, smoking status, history of diabetes, cardiovascular disease, use of angiotensin-converting enzyme inhibitor or angiotensin II receptor blocker medications, serum total albumin, estimated glomerular filtration rate, natural log-transformed proteinuria, and cause of kidney disease

**Table 5:** Added predictive ability for death and ESKD with different carbamylation biomarkers

	IDI	*p* value	Continuous NRI	*p* value
**Death**				
Carbamylated albumin	0.010 (-0.004-0.032)	0.08	0.495 (0.112–0.640)	0.04
Homocitrulline	0.010 (0.000 0.036)	0.08	0.519 (0.310–0.726)	0.03
**ESKD**				
Carbamylated albumin	0.015 (0.001–0.031)	0.04	0.589 (-0.452-0.633)	0.11
Homocitrulline	0.017 (-0.002-0.034)	0.06	0.544 (0.373–0.625)	0.02

The NRI corresponds to the weighted value (1/2 NRI (> 0)). The predictive value is calculated upon adding each biomarker to a fully adjusted base model

Base model is stratified by center and adjusts for age, sex, race, and ethnicity, systolic blood pressure, body mass index, smoking status, history of diabetes, cardiovascular disease, use of angiotensin-converting enzyme inhibitor or angiotensin II receptor blocker medications, serum total albumin, estimated glomerular filtration rate, natural log-transformed proteinuria, and cause of kidney disease

*Abbreviations*: *ESKD* End stage kidney disease, *IDI* Integrated discrimination improvement, *NRI* Net reclassification index

## Discussion

In this prospective cohort study of patients with CKD, we set out to compare the prognostic performance of two commonly utilized circulating markers of protein carbamylation. Protein carbamylation has emerged as a novel, mechanistically driven risk factor for adverse clinical outcomes in CKD that is of particular interest as it appears to be modifiable [29–31]. Studies have used a variety of assays to assess carbamylation, but their predictive performance has never been compared, limiting the ability to summarize and contrast findings, and to optimally plan future studies. As expected, in the present study we found that C-Alb and HCit were each independently associated with a higher risk for all-cause mortality and CKD progression, corroborating several prior studies [19–21, 32]. We now also show that both biomarkers demonstrated remarkably similar risk associations to each other when utilized concomitantly. The direction and the order of magnitude of the point estimates for each of the markers and respective outcomes were consistent though not identical (most notably for the ESKD outcome). Nevertheless, the 95% confidence intervals around each of the point estimates overlapped considerably, highlighting their similarities. The two markers appeared to also provide similar modest incremental predictive information when added to base models that included standard risk factors for death or CKD progression. Such findings indicate that HCit and C-Alb perform similarly when predicting important clinical outcomes.

It is well reported that levels of C-Alb or HCit differ significantly between individuals who experience meaningful clinical outcomes and those who do not (e.g., mortality, CKD progression, cardiovascular events, even anemia and erythropoietin resistance) [1]. Furthermore, the incremental predictive value of these markers in addition to established risk factors has been shown in preliminary reports [20]. The similar performance of these two markers in our study suggests broad-based comparisons across studies using either marker are possible. Clinical outcome studies in CKD reporting on associations with either assay are likely providing similarly useful information on the risks of carbamylation burden. Importantly, free HCit is a commonly reported analyte measured on metabolomic platforms and the numerous metabolomic studies and databases currently containing HCit data could potentially be utilized to answer key questions in carbamylation science and advance the field rapidly. Future research is needed to determine if any of the differences between C-Alb and HCit observed in our study would result in meaningful differences if applied clinically (e.g., if any differences could influence prospective clinical decision-making).

Upon categorizing the study population into quartiles for each of the two different carbamylation biomarkers, the corresponding concomitant clinical laboratory data for the population appeared similar but not identical. While BUN levels were nearly identical, for example, in the highest C-Alb and HCit groups, the eGFR and proteinuria in these same groups differed with the high HCit group showing worse values (i.e., lower eGFR and higher proteinuria). Indeed, the small molecule HCit was more strongly correlated with eGFR than C-Alb; thus, perhaps HCit is a more sensitive marker of eGFR, and this may have driven some of our findings despite statistical adjustments for eGFR made throughout. Regardless, HCit’s predictive and prognostic performance in models, compared to C-Alb, did not appear different. The inconsistent adjusted associations between the carbamylation markers and other variables shown in Table 2 warrant additional study. While competitive glycation may impact carbamylation levels in diabetics [33], age or race and ethnicity differences are less readily explained. As these associations were not adjusted for multiple testing, they should be interpreted cautiously and require further validation.

In a small ancillary study of 45 hemodialysis patients, investigators reported that protein bound as well as “total” (free and protein bound) HCit levels correlated with measures of albumin carbamylation [22]. Our study corroborates such expected positive correlation between the different carbamylation measures yet provides several new insights. The clinical prediction modelling presented herein using both markers from the same time point is the greatest advancement. In addition to a significantly larger sample size from a distinct patient population (non-dialysis CKD), our study also demonstrates that *free* HCit measures alone correlated robustly to the carbamylation of the most abundant circulating protein, albumin. Free HCit is the form captured by metabolomic platforms such as the one employed in this study, and it does not require steps to cleave peptide bonds (required to measure total HCit), or sample dialysis and protein precipitation (used to remove free HCit and yield only protein bound measures) [34]. Free HCit measures, which are commonly available in existing metabolomic databases, performed remarkably similar to C-Alb. The significantly greater correlation we observed between HCit and C-Alb vs. the prior report may be a function of our use of free HCit alone, sample size, differences in patient populations, or differences in assay techniques.

While statistically correlated (correlation coefficient 0.64), the markers do not appear related to each other in a strict 1:1 manner. Moreover, each marker demonstrated strong correlations to BUN levels, though, again, not with a 1:1 relationship. It is interesting to recall that the correlation reported between serum glucose and glycated hemoglobin (HbA_1c_), the most widely used surrogate marker for time-averaged glucose to guide diabetes management, is 0.42–0.58 [35]. Even at such correlation strengths, HbA_1c_ is instrumental in determining the long-term clinical risks of fluctuating glucose levels [36]. It is likely that carbamylated proteins with long half-lives similarly better depict time-averaged urea exposure than single blood urea measurements which fluctuate significantly in relation to recent diet, hydration, volume, circulatory status, medications, acute GFR changes, and catabolic state [37, 38]. Furthermore, carbamylation occurs through non-uremic processes (e.g., myeloperoxidase catalyzed oxidation of thiocyanate derived from diet and smoking) [15] and can be exacerbated by amino acid deficiencies from nutritional imbalance, protein-energy wasting, or other means [17]. The half-life of the respective markers’ un-carbamylated substrate (lysine and albumin), differ considerably on the order of hours for lysine and weeks for albumin. Nevertheless, the ongoing release of protein bound HCit to free HCit during protein degradation, may explain why the 2 markers appeared to provide similar information. Presenting HCit as a ratio to total lysine has also been proposed [14, 15], and we provide this analysis in the supplement to show overall results were not significantly changed using either HCit alone or as a ratio to lysine. The incremental prediction value added to the fully adjusted risk models was remarkably similar for either marker, potentially showing the *application* of these markers could confer similar utility despite any differences observed in their associations to clinical variables and outcomes.

To this end, we must acknowledge that well-conducted epidemiological studies have shown that traditional biomarkers such as eGFR and proteinuria, plus easily available clinical parameters, perform very well in terms of identifying CKD patients at high risk of future adverse outcomes (e.g., the baseline C-statistic using traditional markers to predict CKD progression was ~ 0.9 from a previously published study in CRIC) [39]. As the authors of this prior study conclude, it is unlikely that such a high C-statistic can be significantly improved on or even needs improving. Indeed, we observed only modest changes in C-statistics when carbamylation markers were added, but the key finding for this report is that the magnitude of change was similar for each marker. Beyond C-statistics, which can have several limitations [40], carbamylation markers can provide important pathophysiological information which can lead to novel therapeutic targets as well as monitoring of treatment effectiveness. Further, the NRI performance of the two markers appeared to be more clinically meaningful and similar across both.

Our results need to be interpreted in the context of their limitations. We were only able to compare single time point measurements of the biomarkers at baseline without subsequent longitudinal measurements. External validation ideally employing serial measurements to assess performance characteristics remains a future direction. Also, as HCit can exist in both protein-bound and free forms in biological systems, we could not account for relative differences from our metabolomic data that generated only free HCit results for this study. Some previous studies have used protein bound HCit as a biomarker for carbamylation and it is possible our findings may have differed if doing so in this study [14, 15]. Thus generalizability to other HCit based studies requires cautious interpretation based on the assay methods. Further, the objective of this study was to compare the performance of the two biomarkers, rather than provide an accurate estimate of the hazard ratios associated with each. Nevertheless, strengths of our study include the large sample size and use of the CRIC study, the largest prospective cohort study for CKD in the United States offering patients from diverse racial and ethnic backgrounds, and rigorous outcome ascertainment and covariable data.

## Conclusions

In summary, our study suggests that C-Alb and HCit perform similarly in terms of associations with important clinical endpoints in a large CKD cohort. The markers also show similar measures of risk discrimination and reclassification when looking at clinical outcomes. To this end, it appears that C-Alb and HCit are both reasonable markers to employ in carbamylation related studies and could be comparable to one another across studies acknowledging the observed differences reported herein. The many clinical studies with existing metabolomic data including HCit could be readily analyzed to answer carbamylation specific research questions. Future work will need to establish if there is superiority between the markers when looking towards specific clinical applications.

### Supplementary Information

Supplementary Material 1.

## Data Availability

Anonymized and original observational data reported in this paper will be uploaded to the NIDDK Biorepository on publication for public access.

## Abbreviations

CKD: Chronic kidney disease
ESKD: End stage kidney disease
CRIC: Chronic renal insufficiency cohort
C-Alb: Carbamylated albumin
HCit: Homocitrulline
