# Supplementary material for: Comparative CKD risk prediction using homocitrulline and carbamylated albumin: two circulating markers of protein carbamylation
Source: BMC Nephrol. 2024 May 30;25:185. doi: 10.1186/s12882-024-03619-6 (PMC11140876; doi:10.1186/s12882-024-03619-6)
Supplement: Supplementary file 1 — Supplementary Material 1. [file 12882_2024_3619_MOESM1_ESM.docx]

**Supplementary Materials**

Awwad et. al., *Comparative Risk Prediction Using Homocitrulline and Carbamylated Albumin: Two Circulating Markers of Protein Carbamylation*

**Supplementary Table 1-** Baseline characteristics of the study participants according to carbamylated albumin quartiles.

**Supplementary Table 2-** Baseline characteristics of the study participants according to homocitrulline quartiles.

**Supplementary Table 3-** Correlation analysis between carbamylation biomarkers and blood urea nitrogen.

**Supplementary Table 4-** Risk of primary and secondary endpoints by carbamylated albumin with additional adjustments.

**Supplementary Table 5-** Risk of primary and secondary endpoints by homocitrulline with additional adjustments.

**Supplementary Table 6-** Baseline characteristics of the study participants according to homocitrulline /lysine.

**Supplementary Table 7-** Factors associated with levels of homocitrulline/lysine ratio in univariable and multivariable linear regression analysis.

**Supplementary Table 8-** Risk of primary and secondary endpoints by homocitrulline/lysine ratio.

**Supplementary Table 9-** Comparison of C-statistic values with addition of homocitrulline/lysine to a base model.

**Supplementary Table 10-** Comparison of NRI and IDI for homocitrulline/lysine with addition of homocitrulline/lysine to a base model.

**Supplementary Table 11.** Analysis of ESKD risk when death is treated as a competing outcome.

**Supplementary Table 12.** eGFR stratified analysis of the primary and secondary outcomes

**Supplementary Figure 1-** Flow chart of the selection of the study participants.

**Supplementary Figure 2-** Box plots illustrating the distribution of the biomarker values across quartiles.

**Supplementary Figure 3.** Concordance counts across quartiles of carbamylated albumin (C-Alb) and homocitrulline (H-Cit).

**Supplementary Table 1.** Baseline characteristics of the study participants according to carbamylated albumin quartile.

|  | **Carbamylated Albumin** | | | | |
| --- | --- | --- | --- | --- | --- |
|  | **Overall** | **1^st^ quartile** | **2^nd^ quartile** | **3^rd^ quartile** | **4^th^ quartile** |
| n | 1,632 | 408 | 408 | 408 | 408 |
| Age, years | 59 (11.0) | 55 (10.7) | 59 (10.0) | 60 (10.9) | 60 (11.0) |
| **Sex, No (%)** |  |  |  |  |  |
| Female | 702 (43) | 183 (45) | 187 (46) | 161 (40) | 171 (42) |
| **Race/Ethnicity (%)** | | | | | |
| Non-Hispanic White | 700 (43) | 170 (42) | 190 (47) | 176 (43) | 164 (40) |
| Non-Hispanic Black | 690 (42) | 186 (46) | 164 (40) | 166 (41) | 174 (43) |
| Hispanic | 187 (12) | 35 (9) | 41 (10) | 52 (13) | 59 (15) |
| Other | 55 (3) | 17 (4) | 13 (3) | 14 (3) | 11 (3) |
| **Past Medical History, No (%)** | | | | | |
| Hypertension | 1466 (90) | 334 (82) | 368 (90) | 379 (93) | 385 (95) |
| Diabetes | 816 (50) | 180 (44) | 203 (50) | 197 (48) | 236 (58) |
| CHF | 182 (11) | 32 (8) | 37 (9) | 39 (10) | 74 (18) |
| Stroke | 182 (11) | 32 (8) | 44 (11) | 54 (13) | 52 (13) |
| PVD | 123 (8) | 15 (4) | 28 (7) | 27 (7) | 53 (13) |
| Current Smoking | 197 (12) | 54 (13) | 49 (12) | 50 (12) | 44 (11) |
| BMI (mean (SD)) | 32 (7.7) | 34 (7.4) | 33 (7.5) | 32 (7.6) | 31 (7.8) |
| SBP, mmHg (mean (SD)) | 130 (20.9) | 130 (20.9) | 130 (18.9) | 130 (20.6) | 130 (22.7) |
| **Medication use, No (%)** | | | | | |
| Aspirin | 774(48) | 171 (42) | 210 (52) | 183 (45) | 210 (52) |
| Beta blocker | 844 (52) | 187 (46) | 197 (48) | 215 (53) | 245 (61) |
| Statins | 970 (60) | 214 (53) | 246 (60) | 239 (59) | 271 (67) |
| ACE or ARB | 1139 (70) | 247 (61) | 288 (71) | 300 (74) | 304 (75) |
| **Laboratory data** | | | | | |
| Serum creatinine, mg/dL | 2.0 (1.0) | 1.5 (0.5) | 1.7 (0.5) | 2.1 (0.8) | 2.8 (1.3) |
| eGFR ml/min/1.73 m^2^ | 42 (16) | 55 (15) | 46 (13) | 39 (12) | 30 (12) |
| Urinary protein, g/ 24h | 0.17 [0.07, 0.92] | 0.12 [0.06, 0.51] | 0.14 [0.07, 0.83] | 0.21 [0.07, 1.07] | 0.33 [0.10, 1.16] |
| Blood urea nitrogen, mg/dL | 32 (16) | 20 (7) | 26 (8) | 32 (11) | 48 (19) |
| Serum albumin, g/dL | 4.0 (0.4) | 4.1 (0.4) | 4.0 (0.5) | 4.0 (0.4) | 4.0 (0.4) |
| Hemoglobin, g/dL | 13 (1.8) | 14 (1.6) | 13 (1.8) | 13 (1.7) | 12 (1.6) |

Abbreviations: No, number; CHF, congestive heart failure; PVD, peripheral vascular disease; BMI, body mass index (calculated as weight in kilograms divided by height in meters squared); SBP, systolic blood pressure; ACE, angiotensin-converting enzyme inhibitor; ARB, angiotensin II receptor blocker; eGFR, estimated glomerular filtration rate.

Values for continuous variables are presented as mean (SD) or median [interquartile range], unless otherwise noted.

**Supplementary Table 2.** Baseline characteristics of the study participants according to homocitrulline quartiles.

|  | **Homocitrulline** | | | | |
| --- | --- | --- | --- | --- | --- |
|  | **Overall** | **1^st^ quartile** | **2^nd^ quartile** | **3^rd^ quartile** | **4^th^ quartile** |
| n | 1,632 | 408 | 408 | 408 | 408 |
| Age, years | 59 (11.0) | 57 (10.7) | 60 (10.5) | 59 (10.8) | 59 (11.3) |
| **Sex (%)** |  |  |  |  |  |
| Female | 702 (43) | 166 (41) | 171 (42) | 171 (42) | 194 (48) |
| **Race/Ethnicity (%)** | | | | | |
| Non-Hispanic White | 700 (43) | 215 (53) | 198 (49) | 168 (41) | 119 (29) |
| Non-Hispanic Black | 690 (42) | 157 (39) | 159 (39) | 175 (43) | 199 (49) |
| Hispanic | 187 (12) | 25 (6) | 33 (8) | 50 (12) | 79 (19) |
| Other | 55 (3) | 11 (3) | 18 (4) | 15 (4) | 11 (3) |
| **Past Medical History, No (%)** | | | | | |
| Hypertension | 1466 (90) | 324 (80) | 360 (88) | 391 (96) | 391 (96) |
| Diabetes | 816 (50) | 120 (29) | 200 (49) | 223 (55) | 273 (67) |
| CHF | 182 (11) | 21 (5) | 30 (7) | 49 (12) | 82 (20) |
| Stroke | 182 (11) | 29 (7) | 52 (13) | 49 (12) | 52 (13) |
| PVD | 123 (8) | 13 (3) | 27 (7) | 37 (9) | 46 (11) |
| Current Smoking | 197 (12) | 37 (9) | 51 (13) | 61 (15) | 48 (12) |
| BMI (mean (SD)) | 32 (7.7) | 32 (7.2) | 32 (7.7) | 33 (7.8) | 33 (8.0) |
| SBP, mmHg (mean (SD)) | 130 (20.9) | 120 (18.5) | 120 (21.1) | 130 (19.4) | 130 (23.0) |
| **Medication use, No (%)** | | | | | |
| Aspirin | 774(48) | 174 (43) | 183 (45) | 211 (52) | 206 (51) |
| Beta blocker | 844 (52) | 166 (41) | 195 (48) | 222 (55) | 261 (64) |
| Statins | 970 (60) | 192 (47) | 237 (58) | 266 (66) | 275 (68) |
| ACE or ARB | 1139 (70) | 239 (59) | 284 (70) | 317 (79) | 299 (74) |
| **Laboratory data** | | | | | |
| Serum creatinine, mg/dL | 2.0 (1.0) | 1.4 (0.4) | 1.7 (0.4) | 2.0 (0.6) | 2.9 (1.3) |
| eGFR ml/min/1.73 m^2^ | 42 (16) | 58 (14) | 45 (11) | 38 (11) | 27 (11) |
| Urinary protein, g/ 24h | 0.17 [0.07, 0.92] | 0.09 [0.05, 0.20] | 0.13 [0.06, 0.62] | 0.30 [0.08, 1.15] | 0.56 [0.15, 1.87] |
| Blood urea nitrogen, mg/dL | 32 (16) | 20 (7) | 25 (7) | 32 (10) | 49 (19) |
| Serum albumin, g/dL | 4.0 (0.4) | 4.2 (0.4) | 4.1 (0.4) | 4.0 (0.5) | 3.9 (0.5) |
| Hemoglobin, g/ dL | 13 (1.8) | 14 (1.6) | 13 (1.8) | 13 (1.6) | 12 (1.6) |

Abbreviations: No, number; CHF, congestive heart failure; PVD, peripheral vascular disease; BMI, body mass index (calculated as weight in kilograms divided by height in meters squared); SBP, systolic blood pressure; ACE, angiotensin-converting enzyme inhibitor; ARB, angiotensin II receptor blocker; eGFR, estimated glomerular filtration rate. Values for continuous variables are presented as mean (SD) or median (interquartile range), unless otherwise noted.

**Supplementary Table 3.** Correlation analysis between carbamylation biomarkers and blood urea nitrogen.

|  | Blood Urea Nitrogen | |
| --- | --- | --- |
|  | Pearson correlation coefficient | P value |
| C-Alb | 0.72 | <.001 |
| HCit | 0.77 | <.001 |
| HCit/Lysine | 0.74 | <.001 |

* All values are on the log scale.

**Supplementary Table 4**. Risk of primary and secondary endpoints by carbamylated albumin with additional adjustments.

| **Carbamylated Albumin** | **Hazard Ratio** | | |
| --- | --- | --- | --- |
|  | **Unadjusted** | **Adjusted model*** | **Adjusted model + BUN** |
| **Death** |  |  |  |
| Continuous scale, per 1-SD increase | 1.48 (1.37-1.61) | 1.24 (1.11-1.39) | 1.21 (1.05-1.38) |
| Quartile 1 | *Reference* | *Reference* | *Reference* |
| Quartile 2 | 1.35 (1.01-1.81) | 1.22 (0.90-1.66) | 1.21 (0.89-1.65) |
| Quartile 3 | 1.99 (1.51-2.62) | 1.54 (1.13-2.10) | 1.51 (1.10-2.06) |
| Quartile 4 | 2.99 (2.29-3.90) | 1.90 (1.35-2.66) | 1.75 (1.22-2.49) |
| **ESKD** |  |  |  |
| Continuous scale, per 1-SD increase | 2.06 (1.87-2.27) | 1.44 (1.27-1.63) | 1.26 (1.08-1.47) |
| Quartile 1 | *Reference* | *Reference* | *Reference* |
| Quartile 2 | 1.95 (1.38-2.74) | 1.39 (0.96-2.02) | 1.37 (0.95-1.98) |
| Quartile 3 | 3.11 (2.24-4.31) | 1.84 (1.27-2.67) | 1.65 (1.14-2.41) |
| Quartile 4 | 5.72 (4.18-7.83) | 2.16 (1.47-3.19) | 1.58 (1.04-2.40) |

Abbreviations: ESKD, end stage kidney disease; eGFR, estimated glomerular filtration rate; BUN, blood urea nitrogen.

*Adjusted model is stratified by center and adjusts for age, sex, race, and ethnicity, systolic blood pressure, body mass index, smoking status, history of diabetes, cardiovascular disease, use of angiotensin-converting enzyme inhibitor or angiotensin II receptor blocker medications, serum total albumin, estimated glomerular filtration rate, natural log-transformed proteinuria, and cause of kidney disease.

**Supplementary Table 5**. Risk of primary and secondary endpoints by homocitrulline with additional adjustments.

| **Homocitrulline** | **Hazard Ratio** | | |
| --- | --- | --- | --- |
|  | **Unadjusted** | **Adjusted model*** | **Adjusted model + BUN** |
| **Death** |  |  |  |
| Continuous scale, per 1-SD increase | 1.62 (1.48-1.76) | 1.27 (1.10-1.46) | 1.20 (1.02-1.41) |
| Quartile 1 | *Reference* | *Reference* | *Reference* |
| Quartile 2 | 1.88 (1.38-2.56) | 1.19 (0.86-1.65) | 1.21 (0.87-1.68) |
| Quartile 3 | 2.83 (2.10-3.80) | 1.65 (1.18-2.32) | 1.65 (1.17-2.33) |
| Quartile 4 | 3.98 (2.99-5.29) | 1.89 (1.27-2.81) | 1.72 (1.14-2.58) |
| **ESKD** |  |  |  |
| Continuous scale, per 1-SD increase | 2.85 (2.57-3.16) | 1.59 (1.36-1.86) | 1.37 (1.14-1.65) |
| Quartile 1 | *Reference* | *Reference* | *Reference* |
| Quartile 2 | 3.49 (2.24-5.44) | 1.43 (0.90-2.29) | 1.47 (0.92-2.36) |
| Quartile 3 | 6.46 (4.22-9.89) | 1.83 (1.14-2.95) | 1.76 (1.09-2.85) |
| Quartile 4 | 18.23 (12.11-27.44) | 2.92 (1.76-4.83) | 2.35 (1.40-3.94) |

Abbreviations: ESKD, end stage kidney disease; eGFR, estimated glomerular filtration rate; BUN, blood urea nitrogen.

*Adjusted model is stratified by center and adjusts for age, sex, race, and ethnicity, systolic blood pressure, body mass index, smoking status, history of diabetes, cardiovascular disease, use of angiotensin-converting enzyme inhibitor or angiotensin II receptor blocker medications, serum total albumin, estimated glomerular filtration rate, natural log-transformed proteinuria, and cause of kidney disease.

**Supplementary Table 6.** Baseline characteristics of the study participants according to homocitrulline /lysine.

|  | **Homocitrulline/Lysine Ratio** | | | | |
| --- | --- | --- | --- | --- | --- |
|  | **Overall** | **1st quartile** | **2nd quartile** | **3rd quartile** | **4th quartile** |
| n | 1,632 | 408 | 408 | 408 | 408 |
| Age, years | 59 (11.0) | 57 (10.5) | 60 (10.5) | 59 (11.1) | 59 (11.3) |
| **Sex (%)** |  |  |  |  |  |
| Female | 702 (43) | 162 (40) | 166 (41) | 175 (43) | 199 (49) |
| **Race/Ethnicity (%)** | | | | | |
| Non-Hispanic White | 700 (43) | 226 (55) | 190 (47) | 167 (41) | 117 (29) |
| Non-Hispanic Black | 690 (42) | 139 (34) | 161 (40) | 183 (45) | 207 (51) |
| Hispanic | 187 (12) | 28 (7) | 41 (10) | 45 (11) | 73 (18) |
| Other | 55 (3) | 15 (4) | 16 (4) | 13 (3) | 11 (3) |
| **Past Medical History, No (%)** | | | | | |
| Hypertension | 1466 (90) | 327 (80) | 361 (89) | 385 (95) | 393 (96) |
| Diabetes | 816 (50) | 127 (31) | 194 (48) | 227 (56) | 268 (66) |
| CHF | 182 (11) | 22 (5) | 32 (8) | 47 (12) | 81 (20) |
| Stroke | 182 (11) | 30 (7) | 44 (11) | 49 (12) | 59 (14.5) |
| PVD | 123 (8) | 10 (3) | 30 (7) | 35 (9) | 48 (11.8) |
| Current Smoking | 197 (12) | 32 (8) | 41 (10) | 68 (17) | 56 (13.7) |
| BMI (mean (SD)) | 32 (7.7) | 32 (7.1) | 32 (7.7) | 33 (8.2) | 32 (7.7) |
| SBP, mmHg (mean (SD)) | 130 (20.9) | 120 (20.0) | 120 (18.7) | 130 (20.3) | 130 (23.0) |
| **Medication use, No (%)** | | | | | |
| Aspirin | 774 (48) | 170 (42) | 198 (49) | 203 (50) | 203 (50) |
| Beta blocker | 844 (52) | 169 (41) | 200 (49) | 226 (56) | 249 (62) |
| Statins | 970 (60) | 205 (50) | 234 (58) | 258 (64) | 273 (67) |
| ACE or ARB | 1139 (70) | 241 (59) | 296 (73) | 308 (76) | 294 (73) |
| **Laboratory data** | | | | | |
| Serum creatinine, mg/dL | 2.0 (1.0) | 1.4 (0.3) | 1.7 (0.4) | 2.0 (0.6) | 2.9 (1.3) |
| eGFR ml/min/1.73 m^2^ | 42 (16) | 58 (14) | 46 (11) | 38 (11) | 27 (11) |
| Urinary protein, g/ 24h | 0.18 [0.07, 0.92] | 0.09 [0.05, 0.20] | 0.14 [0.06, 0.73] | 0.27 [0.08, 1.08] | 0.55 [0.14, 1.87] |
| Blood urea nitrogen, mg/dL | 32 (16) | 20 (7) | 26 (8) | 32 (10) | 49 (19) |
| Serum albumin, g/dL | 4.0 (0.4) | 4.2 (0.3) | 4.1 (0.4) | 4.0 (0.5) | 3.9 (0.5) |
| Hemoglobin, g/dL | 13 (1.8) | 14 (1.6) | 13 (1.7) | 13 (1.6) | 12 (1.6) |

Abbreviations: No, number; CHF, congestive heart failure; PVD, peripheral vascular disease; BMI, body mass index (calculated as weight in kilograms divided by height in meters squared); SBP, systolic blood pressure; ACE, angiotensin-converting enzyme inhibitor; ARB, angiotensin II receptor blocker; eGFR, estimated glomerular filtration rate.

Values for continuous variables are presented as mean (SD) or median (interquartile range), unless otherwise noted.

**Supplementary Table 7.** Factors associated with levels of homocitrulline/lysine ratio in univariable and multivariable linear regression analysis.

|  | **Univariable analysis** | | **Multivariable analysis** | |
| --- | --- | --- | --- | --- |
|  | β Coefficient | P Value | β Coefficient | P Value |
| Age (per year) | 0.01 | 0.007 | -0.01 | 0.000 |
| Female | 0.11 | 0.037 | 0.05 | 0.133 |
| Black Race | 0.38 | 0.000 | 0.17 | 0.000 |
| Hispanic | 0.61 | 0.000 | 0.13 | 0.023 |
| Other race | 0.09 | 0.000 | 0.05 | 0.566 |
| Diabetes | 0.53 | 0.000 | 0.07 | 0.040 |
| CVD | 0.34 | 0.000 | 0.06 | 0.070 |
| Systolic blood pressure (per mmHg) | 0.01 | 0.000 | 0.00 | 0.277 |
| Body mass index (per 1 unit) | 0.00 | 0.353 | -0.01 | 0.003 |
| Smoking Status | 0.15 | 0.053 | 0.09 | 0.071 |
| eGFR (per mL/min/1.73m^2^) | -0.05 | 0.000 | -0.02 | 0.000 |
| Urinary protein (per g/ 24h) | 0.21 | 0.000 | 0.00 | 0.928 |
| Serum albumin (per g/dL) | -0.54 | 0.000 | -0.09 | 0.027 |
| Hemoglobin (per g/dL) | -0.24 | 0.000 | -0.05 | 0.000 |
| Blood urea nitrogen (per mg/dL) | 1.60 | 0.000 | 0.93 | 0.000 |

**^a^**Multivariable model includes all other variables in the table.

**Supplementary Table 8**. Risk of primary and secondary endpoints by homocitrulline/lysine ratio.

| **Homocitrulline/lysine** | **Hazard Ratio** | | |
| --- | --- | --- | --- |
|  | **Unadjusted** | **Adjusted model*** | **Adjusted model + BUN** |
| **Death** |  |  |  |
| Quartile 1 | *Reference* | *Reference* | *Reference* |
| Quartile 2 | 1.93 (1.40-2.65) | 1.36 (0.97-1.91) | 1.37 (0.98-1.91) |
| Quartile 3 | 2.98 (2.20-4.03) | 2.03 (1.44-2.86) | 2.03 (1.44-2.87) |
| Quartile 4 | 4.55 (3.40-6.09) | 2.49 (1.68-3.69) | 2.31 (1.54-3.46) |
| **ESKD** |  |  |  |
| Quartile 1 | *Reference* | *Reference* | *Reference* |
| Quartile 2 | 3.79 (2.40-5.99) | 1.68 (1.03-2.73) | 1.63 (1.00-2.65) |
| Quartile 3 | 7.29 (4.71-11.30) | 1.99 (1.22-3.23) | 1.90 (1.17-3.09) |
| Quartile 4 | 20.25 (13.25-30.95) | 3.28 (1.96-5.51) | 2.57 (1.50-4.38) |

Abbreviation: ESKD, end stage kidney disease; eGFR, estimated glomerular filtration rate; BUN, blood urea nitrogen.

*Adjusted model is stratified by center and adjusts for age, sex, race, and ethnicity, systolic blood pressure, body mass index, smoking status, history of diabetes, cardiovascular disease, use of angiotensin-converting enzyme inhibitor or angiotensin II receptor blocker medications, serum total albumin, estimated glomerular filtration rate, natural log-transformed proteinuria, and cause of kidney disease.

**Supplementary Table 9.** Comparison of C-statistic values with addition of homocitrulline/lysine to a base model.

| **C-statistic** | **Base model** | **Base model+ Homocitrulline/lysine** |
| --- | --- | --- |
| Death | 0.723 (0.704, 0.741) | 0.726 (0.708, 0.745) |
| ESKD | 0.830 (0.818, 0.842) | 0.836 (0.825, 0.848) |

Abbreviation: ESKD, end stage kidney disease, the biomarker in the model is continuous. Base model is stratified by center and adjusts for age, sex, race, and ethnicity, systolic blood pressure, body mass index, smoking status, history of diabetes, cardiovascular disease, use of angiotensin-converting enzyme inhibitor or angiotensin II receptor blocker medications, serum total albumin, estimated glomerular filtration rate, natural log-transformed proteinuria, and cause of kidney disease.

| **Base model** | **IDI** | **Continuous NRI** |
| --- | --- | --- |
| **Death** | | |
| HCit/Lysine | 0.009 (0.001, 0.032), p value= 0.02 | 0.516 (0.430, 0.693), p value= 0.01 |
| **ESKD** | | |
| HCit/Lysine | 0.015 (0.001, 0.028), p value= 0.02 | 0.531 (-0.499, 0.583), p value= 0.23 |

**Supplementary Table 10.** Comparison of NRI and IDI for homocitrulline/lysine with addition of homocitrulline/lysine to a base model.

Abbreviation: IDI, Integrated discrimination improvement; NRI, Net reclassification index. The NRI corresponds to the weighted value (1/2 NRI (>0)) as mentioned in Pancina et al.(2011)

**Supplementary Table 11.** Analysis of ESKD risk when death is treated as a competing outcome.

|  | Carbamylated albumin | | Homocitrulline | |
| --- | --- | --- | --- | --- |
|  | Unadjusted | Adjusted | Unadjusted | Adjusted |
| Continuous scale  (Per SD increase) | 1.88 (1.71-2.08) | 1.28 (1.10-1.49) | 2.53 (2.28-2.81) | 1.43 (1.21-1.70) |
| Quartile 1 | *Reference* | *Reference* | *Reference* | *Reference* |
| Quartile 2 | 1.91 (1.36-2.68) | 1.32 (0.93-1.88) | 3.32 (2.15-5.13) | 1.57 (1.00-2.46) |
| Quartile 3 | 2.83 (2.05-3.90) | 1.52 (1.06-2.17) | 5.61 (3.70-8.52) | 1.52 (0.95-2.44) |
| Quartile 4 | 4.83 (3.54-6.60) | 1.78 (1.21-2.62) | 14.58 (9.79-21.72) | 2.61 (1.58-4.30) |

Adjusted model is stratified by center and adjusts for age, sex, race, and ethnicity, systolic blood pressure, body mass index, smoking status, history of diabetes, cardiovascular disease, use of angiotensin-converting enzyme inhibitor or angiotensin II receptor blocker medications, serum total albumin, estimated glomerular filtration rate, natural log-transformed proteinuria, and cause of kidney disease

**Supplementary Table 12. eGFR stratified analysis of the primary and secondary outcomes**

| eGFR strata  by outcome | **Adjusted HR** | |
| --- | --- | --- |
|  | **C-Alb** | **HCit** |
| **Death** |  |  |
| eGFR < median | 1.13 (1.01-1.27) | 1.16 (1.00-1.34) |
| eGFR > median | 1.68 (1.36-2.08) | 1.45 (1.13-1.84) |
| **ESKD** |  |  |
| eGFR < median | 1.78 (1.58-2.01) | 2.08 (1.80-2.41) |
| eGFR > median | 1.90 (1.40-2.59) | 1.68 (1.20-2.34) |

Abbreviations: HR, hazard ratio; eGFR, estimated glomerular filtration rate; ESKD, end stage kidney disease). Cutoff was chosen to represent the population eGFR median (40.8 ml/min/1.73 m^2^). Each model adjusted for age, sex, race/ethnicity, systolic blood pressure, body mass index, smoking status, diabetes, cardiovascular disease, use of angiotensin-converting enzyme (ACE) inhibitor or angiotensin II receptor blocker (ARB) medications, serum total albumin, natural log-transformed proteinuria, and cause of kidney disease.

**Supplementary Figures**

**
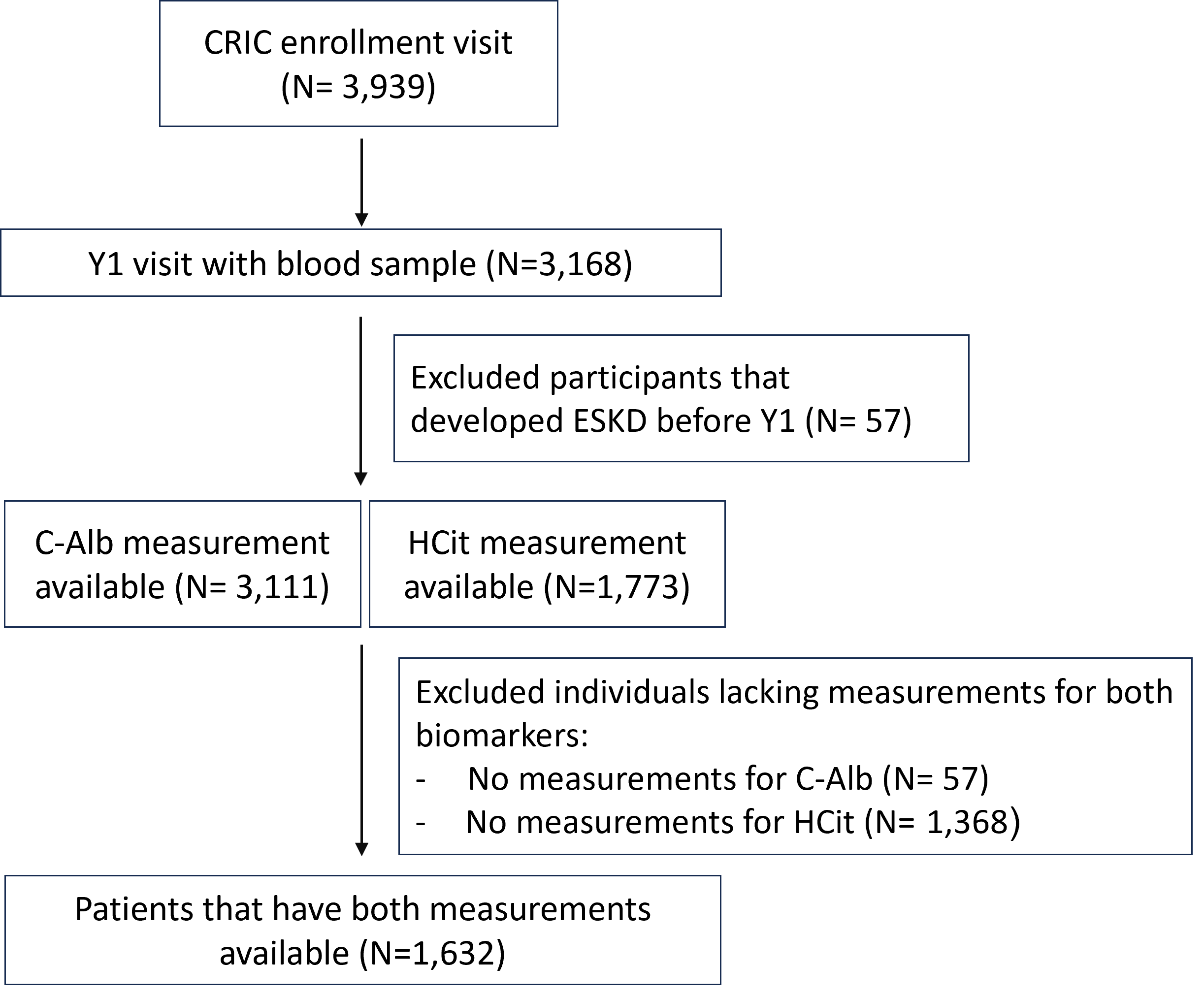
**

**Supplementary Figure 1.** Flow chart of the selection of the study participants.

**
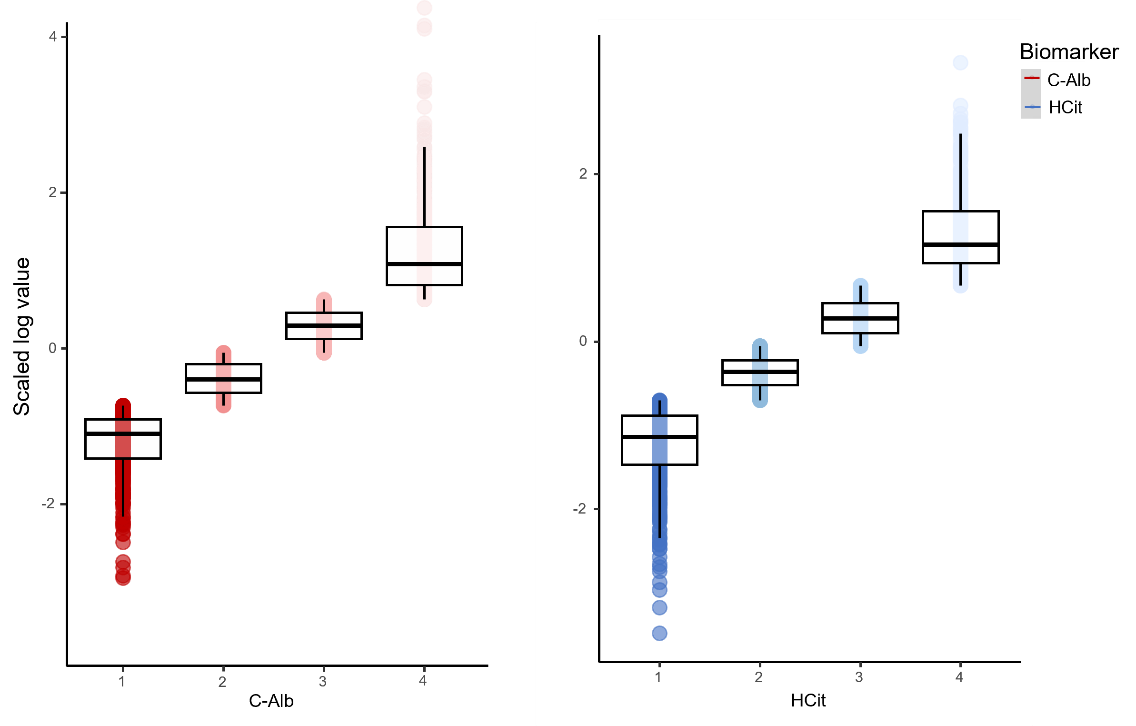
**

**Supplementary Figure 2.** Box plots illustrating the distribution of the biomarker values across quartiles. The x axis is the biomarkers’s quartile, the y axis is the scaled log values. The medians and interquartile ranges of each quartile demonstrate a comparable pattern. We conducted Welch Two Sample t-tests to compare the mean values between corresponding quartiles of C-Alb and HCit. The results indicate no significant difference in mean values between the corresponding quartiles of the two biomarkers (1st (p value = 0.17), 2nd (p value= 0.09), 3rd (p value = 0.60), and 4th (p value = 0.93) quartiles). Overall, the analysis suggests that the biomarkers' behavior across quartiles is largely comparable.


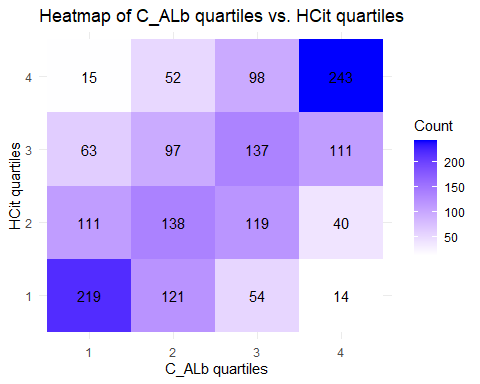


**Supplementary Figure 3.** Concordance counts across quartiles of carbamylated albumin (C-Alb) and homocitrulline (H-Cit). The sum of the numbers in each row corresponds to the total number of individuals in that quartile of HCit. Similarly, the sum of the numbers in each column gives us the total number of individuals in that quartile of C-Alb.
